# Liver Conditions Specific to Pregnancy: Optimizing Management and Outcomes

**DOI:** 10.5152/tjg.2026.26024

**Published:** 2026-04-08

**Authors:** Alexandra Frolkis, Ilkay Ergenc, Michael A. Heneghan

**Affiliations:** 1 Division of Gastroenterology and Hepatology, Department of Medicine, University of Calgary, Calgary, Canada; 2 The Roger Williams Institute of Liver Studies, King’s College Hospital, London, UK; 3 King’s College School of Immunology and Microbial Sciences, Faculty of Life Sciences and Medicine, London, UK

**Keywords:** Fetal outcomes, hepatic function, liver disease, multidisciplinary care, pregnancy

## Abstract

Pregnancy is accompanied by complex physiological adaptations that influence hepatic metabolism, bile transport, coagulation, and immune tolerance, which may precipitate the development of pregnancy-specific liver disorders. Gestational liver disease comprises a distinct group of conditions such as hyperemesis gravidarum (HG), intrahepatic cholestasis of pregnancy (ICP), acute fatty liver of pregnancy (AFLP), and the spectrum of preeclampsia that includes hepatic involvement and Hemolysis, elevated liver enzymes, and low platelets (HELLP) syndrome. Although heterogeneous in presentation and pathophysiology, these disorders share the potential for significant maternal and fetal morbidity and mortality. Early recognition and hepatology-guided interpretation of abnormal liver biochemistry during pregnancy are critical to differentiate gestational liver disease from underlying chronic liver disease and to identify conditions that require urgent intervention.

Management of gestational liver disease requires coordinated, multidisciplinary care focused on maternal stabilization, appropriate disease-specific therapy, and carefully timed delivery according to both maternal and fetal risk. Treatment strategies range from supportive and nutritional management for HG, biochemical and symptom control with ursodeoxycholic acid for ICP, and urgent delivery and intensive supportive care for AFLP to delivery with antihypertensive and anticonvulsant therapy for hypertensive disorders and HELLP syndrome.

Although each condition poses unique diagnostic and therapeutic challenges, complications associated with gestational liver disease in pregnancy may be reduced through proactive management and multidisciplinary involvement. This review summarizes current evidence on the pathophysiology, diagnostic evaluation, management strategies, and pregnancy outcomes associated with pregnancy-specific liver disorders.

**Main Points**

- Pregnancy-specific liver disorders include hyperemesis gravidarum, intrahepatic cholestasis of pregnancy, acute fatty liver of pregnancy, and the spectrum of hypertensive disorders that include hepatic involvement of preeclampsia and HELLP syndrome. Each is associated with overlapping biochemical abnormalities yet distinct diagnostic and therapeutic requirements.
- Gestational liver diseases are collectively associated with substantial maternal and fetal morbidity and mortality, including coagulopathy, hepatic failure, preterm birth, fetal growth restriction, and stillbirth, highlighting the importance of timely diagnosis and intervention.
- Early identification of abnormal liver tests, application of disease-specific diagnostic criteria, and trimester-based risk stratification enable targeted management and appropriate delivery planning across the spectrum of gestational liver diseases.
- Optimal outcomes for both mother and fetus rely on coordinated multidisciplinary care integrating obstetric, hepatology, anaesthetic, and neonatal expertise.

## Introduction

Pregnancy induces complex physiological changes aimed at supporting placental perfusion and fetal development.^1^ These changes include a marked increase in estrogen and progesterone levels, which leads to peripheral vasodilation with reduced vascular resistance, increased cardiac output, and up to a 50% expansion in plasma volume.^2^ Pregnancy results in a procoagulant profile, with increased clotting factors and reduced anticoagulant activity. Physiologic adaptations influence hepatic metabolism and bile transport activity. Immunologic adaptations allow for tolerance of the fetus, shifting the immune response toward a Th2-dominant, anti-inflammatory state.^3^ Although these mechanisms support normal fetal development, they may result in liver dysfunction during pregnancy.

**Figure 1.**
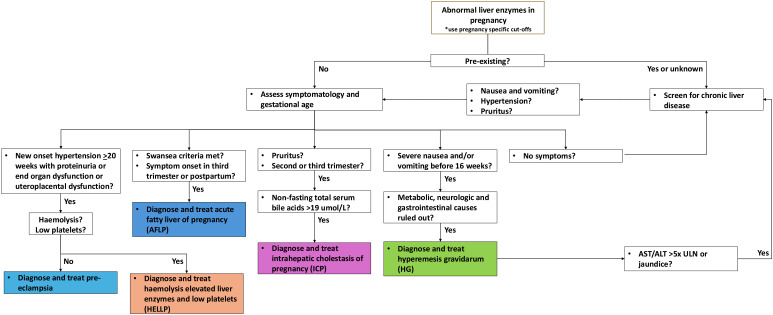
Approach to abnormal liver enzymes in pregnancy. Hypertension defined as systolic blood pressure >140 mmHg and/or diastolic > 90 mmHg on at least 2 occasions measured 4 hours apart in a previous normotensive woman at >20 weeks’ gestation; Swansea criteria are used for diagnosis, with the presence of at least 6 of the following in the absence of an alternative explanation: vomiting, abdominal pain, polydipsia/polyuria, encephalopathy, coagulopathy (prothrombin time > 14 seconds), elevated bilirubin (>0.8 mg/dL), hypoglycemia (<72 mg/dL), elevated ammonia (>27.4 mg/dL), ascites or bright liver on ultrasound, microvesicular steatosis on liver biopsy, elevated transaminases (AST or ALT >42 IU/L), renal impairment (creatinine >1.7 mg/dL), elevated urate (>5.7 mg/dL), or leukocytosis (>11 × 10^9^/L).

**Figure 2.**
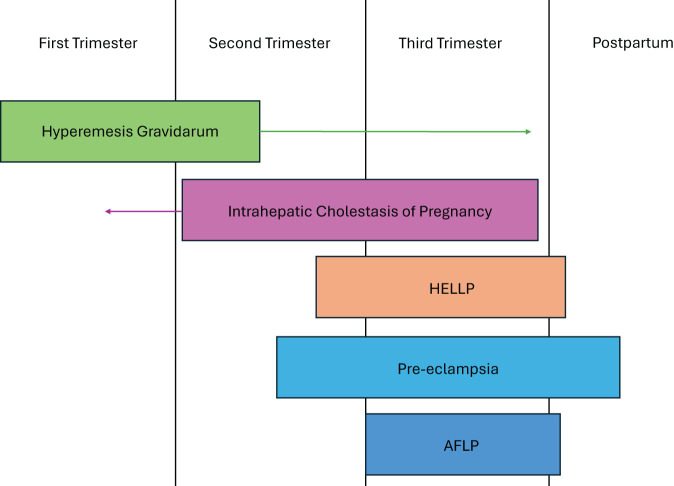
Graphical trimester approach to gestational liver disease. AFLP, acute fatty liver of pregnancy; HELLP, hemolysis, elevated liver enzymes, and low platelets.

Pregnancy may unmask chronic liver disease or give rise to pregnancy-specific hepatic disorders (Figure 1). These include hyperemesis gravidarum (HG), intrahepatic cholestasis of pregnancy (ICP), acute fatty liver of pregnancy (AFLP), and hepatic manifestations within the hypertensive spectrum, including preeclampsia and HELLP syndrome (Figure 2).

Gestational liver diseases are associated with substantial maternal and fetal morbidity and mortality, leading to complications such as coagulopathy, hepatic failure, preterm birth, fetal growth restriction, and, in severe cases, maternal or perinatal death. Early detection and timely management through a multidisciplinary approach are therefore essential to optimize outcomes. Although several high-quality obstetric and hepatology reviews exist, practical guidance on interpreting abnormal findings of the liver tests, differentiating overlapping syndromes, and integrating hepatology into multidisciplinary pregnancy care lacks a unified framework. The purpose of this review was to provide a hepatology-focused overview of liver disorders specific to pregnancy, with particular emphasis on pathophysiology, diagnostic differentiation, management strategies, delivery timing, and postpartum implications.

### Hyperemesis Gravidarum

Hyperemesis gravidarum is a severe form of nausea and vomiting in pregnancy, affecting 0.3%-3.6% of pregnancies.^4^ It is a diagnosis of exclusion with the differential including neurologic, gastrointestinal, and metabolic causes.^5^ The 2021 international consensus Windsor definition describes HG as the onset of severe nausea and vomiting before 16 weeks of gestation, marked by an inability to eat and/or drink normally, leading to significant limitations in daily activities.^6^ Hyperemesis gravidarum has a significant detrimental effect on mental and physical health, with suicidal ideation reported in up to 50% of the affected individuals and termination of pregnancy reported in up to 10% of the affected individuals.^7^ Symptoms are most severe in the first trimester, which typically resolve by the 20th week in over 90% of pregnancies.^8^ However, 20% of those with HG report symptoms that persist throughout pregnancy.^9^ Hyperemesis gravidarum is associated with small-for-gestational-age fetus, low birthweight, and preterm birth.^10^

Despite extensive study, the exact etiology of HG remains unclear. Genome-wide association studies have identified the relationship between GDF15 and IGFBP7 and HG.^11^ GDF15 is a hormone produced by the placenta and acts on the maternal brainstem, influencing appetite and emetic pathways. Although IGFBP7 has been associated with HG, its mechanism of action in HG is less clear. IGFBP7 modulates insulin-like growth factor and has been shown to decrease appetite in animal models. Both GDF15 and IGFBP7 are dysregulated when symptoms of HG peak but not when symptoms subside.^12^Data support a maternal genetic contribution: daughters frompregnancies complicated by HG have a threefold increased risk of developing HG themselves (3.0% vs. 1.1%), whereas female partners of affected sons do not show higher risk.^13^ Female sex of the fetus is associated with HG, suggesting a hormonal mechanism.^14^ Free beta-human chorionic gonadotropin (HCG) in those with HG is significantly higher compared to matched controls.^15^ Molar pregnancy, gastrointestinal disease, and hyperthyroidism have also been associated with HG.^16^

Biochemical abnormalities are commonly seen in severe presentations of HG, with transaminase elevations reported in up to 50% of individuals with HG.^17^ Hyperemesis gravidarum should always be considered in the differential diagnosis of abnormal liver tests in the first trimester. However, an increase in transaminases of >5× the upper limit of normal should prompt a screen for primary liver disease (the differential diagnosis includes gallstone disease, viral hepatitis, autoimmune hepatitis, drug-induced liver injury, Wilson’s disease, vascular liver disease, and steatotic liver disease).^18^ Table 1 summarizes normal ranges for laboratory values in pregnancy. The exact mechanism is unclear, although both dehydration and starvation have been implicated in transaminase elevation.^5^Jaundice and cholestasis have been reported.^19^^,^^20^Although rare, severe complications of HG include Wernicke’s encephalopathy, electrolyte imbalances, vitamin K deficiency, and ketonuria.^21^^-^^23^

Treatment of HG focuses on controlling nausea and vomiting to restore oral intake, prevent dehydration and malnutrition, and improve maternal quality of life. Guidelines recommend admission to hospital in those who are unresponsive to outpatient management, are dehydrated, have >5% total body weight loss, have a confirmed or suspected comorbidity, and are unable to take medications for the confirmed or suspected comorbidity.^18^ First-line treatment includes doxylamine and pyridoxine, prochlorperazine, promethazine, or chlorpromazine. Second-line treatment includes metoclopramide, domperidone, and ondansetron. Third-line treatment includes steroid use. In addition to antiemetic therapy, nutritional support and rehydration are essential. Thiamine supplementation should be given either orally or parenterally to avoid Wernicke’s encephalopathy, particularly in patients with abnormal findings of liver tests.^24^ To reduce morbidity, enteral or parenteral nutrition should be considered for pateints who are not responsive to antiemetic therapy and who exhibit sustained weight loss and nutritional deficiencies.^25^^,^^26^

### Intrahepatic Cholestasis of Pregnancy

Intrahepatic cholestasis of pregnancy is the most common gestational liver disease with a global pooled incidence of 2.9%.^27^ It typically presents in the third trimester of pregnancy, although it is less common in the second trimester. Symptoms may rarely develop in the first trimester.^28^ It is characterized by pruritus with elevated serum bile acid levels (>19 umol/L) and increased risk of adverse fetal outcomes. Pruritus can significantly impact the quality of life of the mother. Adverse fetal outcomes include preterm birth, neonatal admission, and meconium-stained amniotic fluid.^29^The risk of stillbirth is increased in those with peak total serum bile acids (TSBAs) >100 umol/L.^29^ Symptoms should resolve within 1-2 weeks postpartum but may persist up to 4 weeks.

Intrahepatic cholestasis of pregnancy is associated with advanced maternal age, multiple gestation pregnancy, multiparity, diabetes, hypertensive disorders, and^30^ reproductive technology.^31^ Pregnancy-related hormones, and specifically 17β-estradiol and progesterone sulfates, play a role in the pathogenesis of ICP in genetically susceptible individuals (ABCB4 in particular) and in those with previously asymptomatic underlying liver diseases such as hepatitis C, primary biliary cholangitis, and primary sclerosing cholangitis^32^^,^^33^Sulfated progesterone metabolites have been found to inhibit farnesoid X receptor (FXR), resulting in a cholestatic phenotype.^34^Bile salt export pump has been shown to be repressed by 17β-estradiol in the late stages of pregnancy, directly interacting with FXR and contributing to ICP.^35^ Patients with ABCB4, ABCB11, and ATP8B1 variants are at increased risk of developing ICP.^36^^-^^38^

When investigating for ICP, TSBA should be tested in a non-fasting state.^18^^,^^39^ Treatment should be initiated with ursodeoxycholic acid (UDCA) at diagnosis. It is important to note that most assays cannot differentiate endogenous and exogenous bile acids. Measured TSBA may remain elevated during UDCA therapy. The European Association for the Study of the Liver (EASL) guidelines suggest TSBA >40 umol/L in untreated individuals or >100 umol/L in those receiving UDCA treatment to guide management and multidisciplinary delivery recommendations.^18^ Every 2 weeks prior to 32 weeks of pregnancy, TSBA should be measured. Liver enzymes and function should be checked at symptom onset in those with suspected ICP. Although transaminase elevation is commonly seen, jaundice and coagulopathy should raise suspicion of an alternative or concurrent etiology. These include first onset of chronic liver disease and consideration of preeclampsia, HELLP, and AFLP.

Alhough there has been debate in the literature,^40^ society guidelines and systematic reviews have focused on randomized controlled trial data supporting the use of ursodeoxycholic acid (UDCA) to decrease the risk of preterm birth.^18^^,^^41^ The PITCHES trial, which is the largest placebo-controlled trial of UDCA on pregnancy outcomes in ICP, did not demonstrate an association between its use and stillbirth, preterm birth, neonatal death, or neonatal intensive care unit admission.^40^ However, nearly 75% of those in the study had serum bile acids of less than 40 umol/L. When a subgroup analysis of those with serum bile acids >40 umol/L and greater than 100 umol/L was performed, there was not a significant improvement in itch or reduction in bile acids with UDCA.^42^ However, when limited to randomized controlled trials, a systematic review and individual participant data meta-analysis demonstrated an association between UDCA and reduction in stillbirth.^41^ Ursodeoxycholic acid improves pruritus and liver biochemistry. However, its impact on fetal outcomes remains uncertain, and delivery timing based on bile acid levels remains the principal determinant of fetal risk.

Ursodeoxycholic acid has multiple mechanisms of action including stimulation and detoxification of hydrophobic bile acids and stimulation of biliary secretion.^43^ It has been demonstrated to restore the placenta’s ability to carry out vectorial bile acid transfer.^44^ Ursodeoxycholic acid is typically initiated at a dose of 10-15 mg/kg/day to improve liver biochemistry and possibly reduce bile acids and treat pruritus. Cholestyramine may also improve biochemistry and pruritus, although not as effectively as UDCA.^45^The TURRIFIC trial is currently in progress assessing the efficacy of UDCA and rifampicin in those with severe and early onset ICP.^46^ Vitamin K supplementation may be needed particularly in cases of steatorrhea or evidence of coagulopathy.

Deliver timing—rather than biochemical improvement—is the primary determinant of fetal outcomes. In those with TSBA > 100 umol/L, delivery is recommended from 35 weeks’ gestation.^18^ In those with TSBA < 100, induction should be considered by 39 weeks.^18^ Recurrence is common, ranging from 40% to 90% in subsequent pregnancies, and therefore, pregnancy counseling and planning are crucial.

### Acute Fatty Liver of Pregnancy

Acute fatty liver of pregnancy is a rare obstetric and medical emergency that can be life-threatening if not promptly identified and managed. It presents in the third trimester and early postpartum period. It is characterized by microvesicular fat infiltration, leading to progressive hepatic dysfunction and resulting in liver failure. The estimated incidence is approximately 5 in 100 000 pregnancies.^47^ The Swansea criteria are used for diagnosis, with the presence of at least 6 of the following criteria in the absence of an alternative explanation: vomiting, abdominal pain, polydipsia/polyuria, encephalopathy, coagulopathy (prothrombin time > 14 seconds), elevated bilirubin (>0.8 mg/dL), hypoglycemia (<72 mg/dL), elevated ammonia (>27.4 mg/dL), ascites or bright liver on ultrasound, microvesicular steatosis on liver biopsy, elevated transaminases (AST or ALT >42 IU/L), renal impairment (creatinine >1.7 mg/dL), elevated urate (>5.7 mg/dL), or leukocytosis (>11 x 10^9^/L).^48^ Notably, ketonuria can be absent despite prolonged starvation.^49^ Multifetal pregnancies, nulliparity, younger age, male fetus, and low BMI have been identified as risk factors.^47^^,^^50^

Defects in fatty acid metabolism play a role in pathogenesis. Acute fatty liver of pregnancy is classically associated with fatty acid metabolism defects and, in particular, long-chain 3-hydroxyacyl coenzyme A dehydrogenase (LCHAD) in the fetus.^51^^,^^52^ However, it has also been described in the absence of LCHAD mutation.^53^^,^^54^ Variants in fetoplacental mitochondrial fatty acid oxidation are associated with AFLP.^55^The placenta shares the fetal genetic profile and expresses active mitochondrial fatty-acid oxidation pathways.^56^ Breakdown of these pathways is thought to lead to the accumulation of toxic fatty-acid intermediates, and, in particular, arachidonic acid.^57^ Detailed biochemical analysis of placentas from women with AFLP has demonstrated increased concentrations of fatty acid metabolites that are capable of inducing hepatocyte mitochondrial injury, oxidative stress, apoptosis, and lipid deposition.^57^ These toxic intermediates likely enter the maternal circulation and play an important role in mitochondrial dysfunction leading to acute liver failure.

Maternal mortality has decreased with earlier recognition and aggressive supportive care, with recent estimates ranging from 2% to 10%.^47^^,58^ Stillbirth has been reported in up to 12% of cases.^50^ Disseminated intravascular coagulopathy with hemorrhage, ascites, and acute liver failure are the most common adverse maternal effects.^58^ After prompt identification, urgent delivery and supportive care are necessary for the survival of both mother and fetus. After delivery, clinical recovery is seen within 3-4 days, although a cholestatic phase with rising bilirubin and alkaline phosphatase may persist. If liver function does not rapidly improve with delivery, liver transplantation offers the best chance for survival.^59^ The presence of hepatic encephalopathy and elevated lactate are risk factors for liver transplantation and death. An admission lactate of >2.8 mg/dL has 73% sensitivity and 75% specificity for liver transplantation or death.^60^

Although many with AFLP do not choose to pursue further pregnancies, recurrence has been identified in 3%-21%, highlighting the importance of preconception counseling with a multidisciplinary team of hepatologists, obstetricians, and obstetric internal medicine specialists.^61^^,^^62^

### Hypertensive Disorders of Pregnancy

Hypertensive disorders of pregnancy include chronic hypertension, gestational hypertension, preeclampsia, and eclampsia. Hypertension is a leading cause of maternal morbidity, responsible for 14% of maternal deaths.^63^ The hypertensive disorders of pregnancy impact between 6% and 14% of pregnancies.^64^^,^^65^ Preeclampsia results from placental syncytiotrophoblast stress.^66^ During early pregnancy, the placenta remodels uterine vasculature, creating a spiral of arterioles that results in a low-resistance system for oxygen and nutrient exchange between the mother and the fetus.^67^ In some with preeclampsia, impaired spiral arteriole remodeling occurs, resulting in underperfusion and placental ischemia damaging placental villi. This leads to abnormal angiogenesis, endothelial dysfunction, vasoconstriction, and impaired maternal-fetal nutrient exchange.

Preeclampsia exists on a spectrum, which includes HELLP. A recent global meta-analysis identified the worldwide prevalence of preeclampsia at 4.43% and HELLP at 0.39%,^68^ with variation in estimates largely influenced by the criteria used for diagnosis. These hypertensive complications of pregnancy result in significant morbidity and can lead to mortality if not recognized and promptly treated. Fetal complications include intrauterine growth restriction, preterm birth, placental abruption, and stillbirth.^69^Maternal mortality in the Kocaeli University of HDP from 1997 to 2004 identified a maternal mortality of 1.2% from preeclampsia, with all 3 cases complicated by HELLP.^70^

#### Definitions of Preeclampsia and HELLP:

Preeclampsia can present from the second trimester until up to 6 weeks postpartum.^71^ It is defined as systolic blood pressure >140 and/or diastolic > 90 on at least 2 occasions measured 4 hours apart in a previous normotensive woman at >20 weeks’ gestation with the accompaniment of at least 1 of the following:^72^

1. Proteinuria
2. Maternal end-organ dysfunction including neurologic complications of eclampsia, altered mental status, blindness, stroke, severe headache, clonus, or persistent visual scotomata; pulmonary edema; hematologic complications of thrombocytopenia <150,000 mmol/L, disseminated intravascular coagulation, or hemolysis; acute renal injury (creatinine >90 umol/L); or acute liver involvement with alanine aminotransferase (ALT) or AST > 40 IU/L with or without right upper quadrant or epigastric pain.
3. Uteroplacental dysfunction such as placental abruption, angiogenic imbalance, fetal growth restriction, abnormal umbilical artery Doppler waveform analysis, or intrauterine fetal death.

Headache, visual changes, right upper quadrant or epigastric pain, nausea, vomiting, and edema are common symptoms. Risk factors for preeclampsia include nulliparity, multiple gestations, history of preeclampsia, chronic hypertension, pregestational diabetes, thrombophilia including antiphospholipid antibody syndrome, systemic lupus erythematosus, prepregnancy body mass index >30, maternal age of 35 years or older, kidney disease, obstructive sleep apnea, and^73^ reproductive technology.^74^

Liver enzyme abnormalities, and specifically aspartate aminotransferase (AST), are associated with adverse maternal outcomes in preeclampsia.^75^ The AST elevation can be seen in up to 55% of those with preeclampsia.^18^ Liver function abnormalities, although not common in those with preeclampsia, are associated with increased odds of adverse maternal outcomes.^76^

HELLP is defined as the development of hemolysis, elevated liver enzymes, and low platelets in the setting of pregnancy.^77^ This diagnosis is based on the presence of hemolytic anemia with platelets < 100,000/mm^3^, and transaminase elevation greater than twice the upper limit of normal. HELLP is associated with unruptured hepatic and subcapsular hematomas. It typically occurs in the third trimester.^78^ Common presenting signs are epigastric pain, nausea and vomiting, microangiopathic hemolytic anemia, thrombocytopenia, and abnormal liver function tests.^77^^,^^79^

HELLP syndrome is associated with hepatic hematomas. Abdominal ultrasound should be done if there are symptoms suggestive of hepatic hematoma such as abdominal pain, epigastric pain, or right shoulder pain.^18^ Subcapsular rupture has been reported in over 38% of those with HELLP, and capsular rupture occurs in fewer than 2% of cases.^80^^,^^81^ This leads to maternal (32%) and fetal (51%) mortality.^82^

#### Management of Preeclampsia and HELLP:

Delivery of the fetus is definitive treatment for preeclampsia and HELLP. For those who are >37 weeks’ gestation, the risk of maternal adverse outcomes outweighs ongoing gestational benefit to the fetus, and delivery should be initiated after correction of severe hypertension and coagulopathy.^83^ In those with HELLP or preeclampsia with liver involvement, delivery should be considered at >34 weeks after severe hypertension and coagulopathy have been corrected.^18^

Hypertension management includes use of labetalol, nifedipine, or methyldopa in those with non-severe hypertension (systolic blood pressure 140-159 and/or diastolic blood pressure 90-109).^18^ For those with severe hypertension (systolic blood pressure >160 or diastolic blood pressure >110), treatment should occur in a monitored setting with oral labetalol, nifedipine, or methyldopa and use of intravenous therapy when needed.^18^ Magnesium should be administered with severe hypertension to prevent eclamptic seizures.^18^ Dexamethasone or betamethasone can be given if delivery occurs before 35 weeks for fetal lung maturation.^18^

#### Outcomes and Recurrence of Preeclampsia and HELLP:

A large retrospective study of hospital discharges in Canada identified that those with HELLP syndrome had elevated rates of acute renal failure (22.9 per 1000), acute hepatic failure (4.5 per 1000), cerebrovascular morbidity (8 per 1000), cardiac morbidity (11.6 per 1000), DIC (2.6 per 1000), shock (4.1 per 1000), obstetric embolism (2.3 per 1000), severe preeclampsia (55.2 per 1000), and eclampsia (8.6 per 1000).^78^ Stillbirth occurred in 13.5 per 1000 deliveries with HELLP.^78^

The fullPIERS model can be used to identify those at increased risk of adverse outcomes up to 7 days before complications arise and can facilitate in delivery timing. This model includes gestational age, chest pain/dyspnea, oxygen saturation (SpO_2_), platelet count, creatinine, and aspartate transaminase to predict adverse maternal outcomes with an area under the curve of 0.88 (95% confidence interval 0.84-0.92).^84^

Risk of preeclampsia recurrence is increased in subsequent pregnancies. A Swedish registry study demonstrated a 14.7% risk in the second and a 31.9% risk in the third pregnancy in those who had preeclampsia in their first pregnancy.^85^ Low-dose aspirin taken from the end of the first trimester until 36 weeks’ gestation statistically significantly reduces the risk of developing preeclampsia.^86^ In the absence of contraindications, aspirin is recommended for those with HELLP, starting before 16 weeks and continuing at a dose of 150 mg until 36 weeks or when preeclampsia/HELLP is diagnosed.^18^ The ASPRE trial identified that 150 mg of aspirin from 11 to 14 weeks’ gestation until 36 weeks’ gestation resulted in a lower incidence of preterm preeclampsia for those at high risk of development.^87^ In those with low dietary calcium intake, calcium supplementation significantly reduces the risk of preeclampsia; however, the significance of this effect is not seen in those with adequate intake.^88^

#### Distinguishing HELLP from AFLP:

Acute fatty liver of pregnancy can be misdiagnosed as HELLP as they share overlapping features, although are distinct entities (Table 2).^89^ Both AFLP and HELLP syndrome share overlapping third trimester presentations, yet represent distinct pathophysiologic entities. HELLP syndrome is more closely linked to preeclampsia and abnormal placentation, whereas AFLP reflects hepatic microvesicular steatosis related to disordered fatty acid metabolism.^90^^,^^91^ Although both conditions may present with abdominal pain, nausea, and abnormal liver biochemistry, several features favor AFLP. Hypoglycemia, encephalopathy, metabolic acidosis, elevated ammonia, early coagulopathy, and marked renal dysfunction are more characteristic of AFLP and reflect evolving acute liver failure. Elevated lactate and worsening synthetic dysfunction further support this diagnosis. In contrast, HELLP syndrome is defined by microangiopathic hemolysis, thrombocytopenia (typically early), and moderate transaminase elevation in association with hypertension and proteinuria. Severe thrombocytopenia with hemolysis in a hypertensive patient strongly favors HELLP.

Based on findings from a cohort of 134 patients with AFLP and HELLP (67 each), the presence of low fibrinogen (<300 mg/dL), low cholesterol (<220 mg/dL), elevated creatinine (>0.9 mg/mL), and elevated total bilirubin (1 mg/mL) at admission may favor AFLP.^91^ However, disseminated intravascular coagulation may occur in severe HELLP and does not exclude the diagnosis.

The Swansea criteria are widely used for diagnosing AFLP and are sensitive; however, they lack specificity and may overlap with HELLP syndrome or sepsis. Therefore, dynamic clinical assessment and recognition of early liver synthetic failure and hypoglycemia remain critical bedside distinctions. Profound metabolic derangement and early liver failure favor AFLP, whereas hemolysis with severe thrombocytopenia with hypertension favors HELLP.

### Hepatology Follow-Up

Though all gestational liver diseases carry a risk of recurrence in future pregnancies, not all require ongoing hepatology involvement postpartum. Multidisciplinary care is emphasized for patients with complex or severe liver disease during pregnancy and into the postpartum period. In general, persistent liver enzyme or synthetic abnormalities after delivery should prompt hepatology referral to evaluate for underlying chronic liver disease. Hyperemesis gravidarum may be associated with transient liver enzyme elevations in up to 40%-50% of cases, but these typically resolve with clinical improvement and are not linked to chronic liver disease; therefore, no specific hepatology follow-up is required once symptoms and biochemistry normalize.

Intrahepatic cholestasis of pregnancy generally resolves after delivery; however, postpartum reassessment of liver biochemistry is important to confirm normalization and to exclude underlying liver disease if abnormalities persist. Ongoing hepatology involvement is warranted in ICP where pruritus persists beyond 6 weeks postpartum, liver tests remain abnormal, there is a family history suggestive of genetic cholestasis, or another liver disorder (such as viral hepatitis) is identified during evaluation.

Preeclampsia with liver involvement and HELLP syndrome primarily require obstetric and primary care follow-up. While these conditions are associated with increased long-term cardiovascular risk, routine hepatology follow-up is not required once liver tests normalize. In contrast, AFLP represents a severe hepatic insult that may necessitate evaluation for liver transplantation in cases of liver failure, and those requiring transplantation will need lifelong hepatology care.

In all causes of gestational liver disease, there is a risk of recurrence in future pregnancies. Multidisciplinary preconception counseling involving hepatology, obstetric-internal medicine, and obstetrics is recommended to guide on recurrence risk, monitoring, and delivery considerations.

## Conclusion

Gestational liver diseases represent a diverse group of conditions associated with significant maternal and fetal risk, necessitating timely recognition, structured assessment, and coordinated multidisciplinary management. Disorders including HG, ICP, acute fatty liver of pregnancy, HELLP syndrome, and hepatic complications of preeclampsia may present with overlapping biochemical abnormalities, yet require distinct diagnostic and therapeutic approaches. Accurate interpretation of abnormal liver tests in pregnancy therefore requires a hepatology-informed framework that integrates gestational timing, biochemical pattern, clinical features, and risk of hepatic synthetic dysfunction.

Management is centered on maternal stabilization, disease-specific therapy, and the optimization of delivery timing based on gestational age, maternal condition, and fetal risk. Conditions such as AFLP and HELLP require urgent delivery with intensive supportive care, whereas ICP and HG benefit from targeted medical therapies alongside close biochemical and clinical surveillance. Across all gestational liver disorders, serial monitoring throughout pregnancy is critical to ensure early detection of deterioration and to facilitate prompt intervention.

Beyond the acute setting, gestational liver diseases carry implications for recurrence risk, long-term maternal cardiovascular health, and potential underlying hepatic vulnerability. Postpartum reassessment and multidisciplinary preconception counseling are therefore essential components of comprehensive care.

Ultimately, favorable outcomes depend on early recognition, precise diagnostic differentiation, and collaborative multidisciplinary management. A structured hepatology-focused approach allows pregnancies complicated by liver disease to be navigated safely while minimizing the risk of severe maternal and perinatal complications.

## Figures and Tables

**Table 1.** Normal Ranges for Laboratory Values in Pregnancy

	**First Trimester**	**Second Trimester**	**Third Trimester**
Full blood count			
Hemoglobin	11-14 (↓)	10.5-14 (↓)	10.5-14 (↓)
White cell count	6-16 (↑)	6-16 (↑)	6-16 (↑)
Platelet count	150-400 (stable)	150-400 (stable)	150-400 (stable)
Lymphocyte count	1.1-3.6 (↓)	0.9-3.9 (↓)	1.1-3.6 (↓)
Urea and electrolytes			
Blood urea nitrogen	7-12 (↓)	3-13 (↓)	3-11 (↓)
Creatinine	0.4-0.7 (↓)	0.4-0.8 (↓)	0.4-0.8 (↓)
Liver tests			
Total bilirubin (0.3-1.3 mg/dL)	0.1-0.4 (↓)	0.1-0.8 (↓)	0.1-1.1 (↓)
Albumin (3.5-4.6 g/dL)	2.8-3.7 (↓)	2.8-3.7 (↓)	2.8-3.7 (↓)
AST (7-40 IU/L)	10-28 (stable; ULN ↓)	10-29 (stable; ULN ↓)	11-30 (stable; ULN ↓)
ALT (0-40 IU/L)	6-32 (stable; ULN ↓)	6-32 (stable; ULN ↓)	6-32 (stable; ULN ↓)
GGT (11-50 IU/L)	5-37 (stable or ↓)	5-43 (stable or ↓)	3-41 (stable or ↓)
ALP (30-130 IU/L)	32-100 (↑)	43-135 (↑)	133-418 (↑)
Coagulation			
Prothrombin time	10-12 (stable)	10-12 (stable)	10-12 (stable)
Bile acids (non-fasting)	0-19 (stable)	0-19 (stable)	0-19 (stable)

ALT, alanine aminotransferase; ALP, alkaline phosphatase; AST, aspartate aminotransferase; GGT, gamma-glutamyl transferase.

**Table 2.** AFLP versus HELLP

	**HELLP**	**AFLP**
**Clinical**		
Altered level of consciousness	May occur late	+ with ammonia elevation
Hypertension	++	+/−
Polyuria and polydipsia	−	+
Pulmonary edema	−	+
**Laboratory**		
Thrombocytopenia	Early	Late
Coagulopathy	Late*	+
Acidosis	−	+
Acute kidney injury	+/−	++
Abnormal serum liver tests	+	++
Low fibrinogen	−	+
Elevated bilirubin	+/−	++
Hypoglycaemia	−	++
**Pathology**		
Microvesicular steatosis	−	+

If disseminated intravascular coagulation is present, this can be seen additionally in HELLP.AFLP, acute fatty liver of pregnancy; HELLP, hemolysis, elevated liver enzymes, and low platelets

## Data Availability

The data that support the findings of this study are available on request from the corresponding author.
